# Targeting intracellular signaling as an antiviral strategy: aerosolized LASAG for the treatment of influenza in hospitalized patients

**DOI:** 10.1038/s41426-018-0023-3

**Published:** 2018-03-07

**Authors:** Gerhard Scheuch, Sebastian Canisius, Karlheinz Nocker, Thomas Hofmann, Rolf Naumann, Stephan Pleschka, Stephan Ludwig, Tobias Welte, Oliver Planz

**Affiliations:** 1 Bio-Inhalation GmbH, 35285 Gemuenden/Wohra, Hessen Germany; 2 Ventaleon GmbH, 35285 Gemuenden/Wohra, Hessen Germany; 3 Aumapharma LLC, Doylestown, PA 18901 USA; 4 Institute of Medical Virology, Justus Liebig University Giessen, 35392 Giessen, Hessen Germany; 5 Institute of Virology (IVM), Westfaelische Wilhelms-University Muenster, 48149 Muenster, North Rhine-Westphalia Germany; 6 Pneumology Clinic, Medical University Hannover, 30625 Hannover, Lower Saxony Germany; 7 Interfaculty Institute for Cell Biology, Department of Immunology, Eberhard Karls Tuebingen University, 72076 Tuebingen, Baden-Württemberg Germany

## Abstract

Influenza has been a long-running health problem and novel antiviral drugs are urgently needed. In pre-clinical studies, we demonstrated broad antiviral activity of D, L-lysine-acetylsalicylate glycine (LASAG) against influenza virus (IV) in cell culture and protection against lethal challenge in mice. LASAG is a compound with a new antiviral mode of action. It inhibits the NF-κB signal transduction module that is essential for IV replication. Our goal was to determine whether aerosolized LASAG would also show a therapeutic benefit in hospitalized patients suffering from severe influenza. The primary endpoint was time to alleviation of clinical influenza symptoms. The primary analysis was based on the modified intention-to-treat (MITT) population. This included all patients with confirmed influenza virus infection who received at least one treatment. The per protocol (PP) analysis set included all subjects from the MITT population who underwent at least 13 inhalations. In the MITT group, 48 (41.7%) participants (29 LASAG; 19 placebo) had severe influenza. The mean time to symptom alleviation was 56.2 h in the placebo group and 43.0 h in the LASAG group. The PP set consisted of 41 patients (24 LASAG; 17 placebo). The mean time to symptom alleviation in the LASAG group (38.3 h; *P = *0.0365) was lower than that in the placebo group (56.2 h). In conclusion, LASAG improved the time to alleviation of influenza symptoms in hospitalized patients. The present phase II proof-of-concept (PoC) study demonstrates that targeting an intra-cellular signaling pathway using aerosolized LASAG improves the time to symptom alleviation compared to standard treatment.

## Introduction

Influenza is a major acute respiratory disease in humans that causes seasonal epidemics, as well as severe pandemic outbreaks^1^. Vaccination, the prophylactic measure of choice, has limited efficacy^2^. Therapeutic treatments for influenza are therefore urgently needed, especially in the early phases of pandemic outbreaks, when we must rely on antivirals as the only option. Hospitalized patients suffer from severe influenza with remarkably high mortality rates. At present, no existing drugs are licensed for the treatment of severe influenza in hospitalized patients.

Licensed antiviral therapies and many novel approaches under development target the virus directly^3^. Unfortunately, this leads to reduced effectiveness due to adaptive mutations of the viral genome and subsequent development of resistance^4,5^. Our new strategy does not target the virus directly but instead targets an intra-cellular signaling pathway that is essential for viral replication^6–8^. Earlier studies have shown that influenza viruses need to activate cellular signaling factors when crossing intra-cellular barriers^8^. One of these signaling cascades is the NF-κB pathway^9,10^. NF-κB is activated by a wide variety of extracellular agents, including pro-inflammatory cytokines and pathogenic invaders, such as influenza viruses. In previous studies, we and other authors have shown that influenza viruses exploit NF-κB activity for efficient virus production^11,12^. NF-κB acts has pro-apoptotic and pro-viral effects in the context of an influenza virus infection^11^. Inhibition of NF-κB in the host cell interferes with viral replication by blocking an essential nuclear export step and thereby results in reduced virus titers^13–16^.

Acetylsalicylic acid (ASA) and other salicylates are well-known inhibitors of NF-κB activation^17,18^, and they act as specific inhibitors of IKK2 at a low millimolar range^19^. ASA was suspected of inhibiting influenza virus production as early as 1988^20^. One of our earlier studies identified some of the molecular mediators of this inhibition and confirmed the antiviral effects of ASA in vivo^13^. ASA and other salicylates are also inhibitors of cyclooxygenases (COX) and therefore have anti-inflammatory and pain-relieving effects^21^. However, indomethacin, a pure COX inhibitor, showed no inhibition of virus propagation, which is consistent with its lack of effectiveness in inhibiting NF-κB^13^. Furthermore, pre-clinical experiments demonstrated an antiviral effect of ASA only with aerosolized administration to the lung but not when the drug was delivered systemically via oral treatment^13^. Unfortunately, inhalation of pure ASA is not clinically suitable as it may cause respiratory irritation due to ASA’s acidic properties^22,23^. D,L-lysine improves the stability and tolerability of inhaled ASA, which prevents ASA from hydrolyzing and promotes the formation of a salt. The addition of glycine to ASA prevents discoloration and further increases stability. D,L-lysine-acetylsalicylate∙glycine (LASAG) is a water-soluble salt of ASA. It is licensed as Aspirin i.v.®. It is faster-acting and can be administered orally, intravenously or via inhalation. LASAG is a white powder that dissociates readily into ASA and the two amino acids lysine and glycine upon dissolution into aqueous media. Both lysine and glycine are essential amino acids and are considered to have no relevant pharmacodynamic or toxic effects. They present no risk to human health. As LASAG immediately dissociates into ASA, the pharmacodynamics of LASAG are equivalent to those of ASA^24^. LASAG demonstrated superior antiviral effect compared to ASA in cell culture and in a mouse model of influenza virus infection^25^.

The question that therefore arises is whether LASAG has similar antiviral activity in a clinical setting. To answer this question, a PoC study was conducted to assess the benefits of aerosolized LASAG in the treatment of hospitalized patients with severe influenza, with a primary endpoint of time to alleviation of clinical symptoms.

## Materials and methods

### Drugs

Commercial Tamiflu® and Aspirin i.v.® (LASAG) were used for treatment. LASAG is a salt of ASA and two amino acids, glycine, and lysine.

For pre-clinical studies, LASAG (BAY 81–8781; MW = 363.5) and ASA (MW = 180.1) were provided by Bayer HealthCare AG (Wuppertal, Germany). Prior to the experiments, a 10-mM stock solution was prepared by dissolving 10.9 mg of LASAG in 3 ml of BA medium. For ASA, a 10-mM stock solution was prepared by dissolving 5.4 mg of ASA in 1 ml of PBS and incubating the solution for 5 min at 37 °C. The pH was adjusted to 7.4 with 1 N NaOH (Riedel-de-Haën, Germany). For in vivo studies, 3.50 g of LASAG was dissolved in 35 ml of ddH_2_O (10%) immediately prior to the experiments. Oseltamivir-carboxylate was purchased from Toronto Research Chemicals, Canada.

### Virus

Cell culture experiments where conducted with influenza A virus strain, A/Puerto Rico/8/34 (H1N1) with a multiplicity of infection (MOI) of 0.01, as well as A/Regensburg/D6/09 (H1N1pdm09), highly pathogenic avian influenza virus A/FPV/Bratislava/79 (H7N7) and H5N1-subtype avian influenza virus A/mallard/Bavaria/1/2006 (H5N1), all with an MOI of 0.001.

### Progeny virus inhibition assay

A549 cells were infected with different influenza virus strains for 30 min at 37 °C in a 5% CO_2_ atmosphere. After incubation, the virus dilution was aspirated, and the cells were treated with 5 µM LASAG with 0.1 µM oseltamivir-carboxylate or a combination of LASAG and oseltamivir-carboxylate (OC) (0.1 µM OC/1000 µM LASAG; 0.1 µM OC/100 µM LASAG; 0.1 µM OC/10 µM LASAG) for 24 h at 37 °C in 5% CO_2_. The cell culture supernatants were collected to determine the progeny virus titers using the AVICEL® plaque assay, as described previously^26,27^.

### Inoculation of mice

Eight-week-old female C57BL/6 mice (four per group) were anesthetized by intra-peritoneal injection of 200 µl of ketamine/rompun. Equal amounts of a 2% rompun (Bayer; Germany) and a 10% ketamine (Sanofi; Germany) stock solution were mixed with PBS in a 1:10 ratio. Mice were infected intra-nasally with 1.5 × 10^5^ PFU (5 × MLD_50_) of A/FPV/Bratislava/79 (H7N7) diluted in 50 µl of BSS (buffered salt solution) by inoculating 25 µl into each nostril one hour after application of anesthesia. The Institutional Animal Care and Use Committee of Tuebingen approved all animal studies. Mice were sacrificed 24 h post-infection (p.i.), after which the lungs were weighed, transferred into a Lysing Matrix D tube (MP Bio, Germany) and mixed with a 10-fold volume of BSS. Organs were shredded using the FastPrep FP 120 device (Savant, Germany). To remove the cell debris, the homogenates were centrifuged for 15 min at 2000 r.p.m. and the supernatant was collected. The virus titers of the homogenates were determined using the AVICEL® plaque assay^28,29^.

### Western blot analysis

For Western blots, cells were lysed on ice with RIPA lysis buffer (1% (v/v) NP-40, 0.5% (v/v) DOC, 1% (w/v) SDS, 150 mM NaCl, 50 mM Tris pH 8.0, 200 mM pefablock, 5 mg/ml aprotinin, 5 mg/ml leupeptin, 1 mM sodium-vanadate, and 5 mM benzamidine) for 30 min. Cell lysates were cleared by centrifugation and protein yields were estimated using the Bio-Rad protein assay (Bio-Rad Laboratories; Germany). Equal amounts of protein were separated by SDS-polyacrylamide gel electrophoresis and subsequently transferred onto nitrocellulose membranes. Antibodies against pan-JNK1, p-JNK, and p-p38 were purchased from Cell Signaling Technology. Antibodies against IκB were purchased from Santa Cruz Biotechnologies and diluted to 1:500 in blocking buffer. Loading controls were performed with anti-ERK2 that was purchased from Santa Cruz Biotechnologies and diluted to 1:500 in blocking buffer. Protein bands were visualized using a standard enhanced chemo-luminescence reaction.

### Luciferase reporter gene assay

A549 cells were transfected with Lipofectamine 2000 (Invitrogen; Germany) according to a protocol described earlier^30^. The 3xNF-κB–tk construct contains 3 copies of a NF-κB– binding motif cloned upstream of a minimal promoter-driven luciferase gene^15^. The IL-8 luciferase reporter constructs have been described previously^31^. The hIL-6 promoter luciferase construct was obtained from the Belgian Coordinated Collections of Microorganisms (BCCM/LMBP) Plasmid Collection. All promoter reporter gene plasmids were derived from a pGL basic plasmid (Promega). Cells were harvested 16 h after transfection using 200 μl of lysis buffer (50 mM Na-MES at pH 7.8, 50 mM Tris-HCl at pH 7.8, 10 mM dithiothreitol (DTT) and 2% Triton X-100). Luciferase activity levels were determined as previously described^32^ and are presented as relative fold activation ± SEM from three independent transfections.

### Study design and patients

This multicenter, randomized, double-blind, parallel-group, placebo-controlled phase IIa study evaluated inhaled LASAG (three times daily) plus standard-of-care treatment versus placebo (three times daily) plus standard-of-care (SoC) treatment in patients who were recruited from 23 hospitals in seven different countries (Czech Republic, Germany, Latvia, Lithuania, Slovakia, Spain and Romania). The study was performed in patients hospitalized for severe influenza and/or complications of co-morbidities due to an acute influenza virus infection. We excluded patients with an acute need for ICU admission and/or mechanical or non-invasive ventilation, as the administration of LASAG via a smart nebulizer does not currently allow its use in ventilated patients. Standardized influenza assessment tools incorporating both patient symptoms and clinical signs of infection were used to assess severity. As acute ICU admission was an exclusion criterion, scores usually applied to ICU patients with influenza were not used. Patients with a reported duration of illness of less than 120 h were included in the study. Other inclusion criteria included: presence of at least one respiratory symptom (nasal congestion, sore throat, or cough) of any severity and hospital admission due to (suspected) influenza; presence of at least one constitutional symptom (aches/myalgia, fatigue, headache or feverishness/chills/sweats) of any severity and presence of fever (temperature ≥38.0 °C orally, or ≥38.5 °C rectally) at the time of screening. Exclusion criteria included known allergy or hypersensitivity to ASA or LASAG; immunization against influenza virus with a live attenuated virus vaccine 4 weeks prior to the study; and presence of any cancer (hematologic or solid tumor) requiring chemotherapy or radiation therapy. The study protocol was approved first by the Federal Institute for Drugs and Medical Devices (BfArM) in Germany, then by the ethics committees and regulatory authorities for each study center. Participants provided written informed consent.

### Randomization and masking

Randomization numbers were generated centrally using an SAS random generator routine with a 1:1 (LASAG:placebo) ratio. Actual assignment of random numbers was conducted by un-blinded staff member in chronological order of participant enrollment. The blinded vials, provided by un-blinded staff member, were used to fill the AKITA-JET® nebulizer for administration to each participant after randomization.

### Procedures

All patients received the standard treatment available at each study site, including Tamiflu® if prescribed, at the investigators’ discretion. Patients were randomized to two different inhalation treatment groups. The LASAG group received 800 mg of LASAG/4 ml of fill dose, equivalent to 400 mg of ASA/4 ml of fill dose, resulting in an alveolar dose of 45 mg ASA. The placebo group received 4 ml saline solution (0.9% sodium chloride (NaCl)) dissolved in water. Isotonic saline had no metabolic activity and was administered using the same inhalation system. Both patients and investigators were blinded to the treatment. Prior to each inhalation session, the inhalation solution was freshly prepared by an un-blinded study personnel not involved in any other assessments. The AKITA-JET® nebulizer was used to nebulize approximately 100 mg of ASA (roughly 1 ml of the fill dose) with a fixed inhalation lung volume of 800 ml within 96 breaths for each inhalation session. Activaero GmbH (now Vectura GmbH) in Gemuenden, Germany, manufactured the device. The measured delivered dose was 101.7 ± 5.9 mg of ASA. Lung deposition and alveolar deposition were calculated according to the mathematical model developed by Koebrich, resulting in a lung dose of 69.0 mg and an alveolar dose of 44.5 mg^33^. As the inhalations were performed three times daily, this resulted in a total daily alveolar dose of 133.5 mg.

### Outcomes

The primary outcome of this study was the time to alleviation of clinical influenza symptoms. This was defined as the time in hours after the first inhalation of the study drug when at least 5 of 7 clinical influenza symptoms were rated as 0 (not present, i.e., same as before onset of influenza) or 1 (mild) and remained so for at least 24 ± 2 h. A validated influenza symptom questionnaire was used for documentation. Symptoms were only scored when patients were not treated with symptom relief medication (i.e., acetaminophen). The composite symptom score (CSS) based on the influenza symptom questionnaire at baseline was used to identify patients with relevant influenza symptoms (CSS ≥ 14). Patients with a CSS < 14 were considered as having only mild symptoms and were subsequently excluded from further analyses, as they would not represent the intended population of severely affected patients. Secondary outcomes included the time to alleviation of clinical signs, change in daily activity score, and viral load. The time to alleviation of clinical signs was defined as the time in hours after first inhalation of LASAG until resolution of at least 4 out of the following 5 clinical signs: body temperature (°C), oxygen saturation (%) (without supplemental oxygen), respiratory rate (1/minute), heart rate (bpm), and systolic blood pressure (mm HG). Two of the 4 resolution criteria had to be body temperature and oxygen saturation. These resolution criteria had to be maintained for at least 24 ± 2 h without use of symptomatic relief medication (i.e., acetaminophen).

The safety analysis (SA) set included all randomized subjects in the trial who received at least one dose of study drug. The SA focused on the following: adverse events; treatment emergent adverse events (TEAEs), especially TEAEs commonly associated with the use of acetylsalicylic acid (e.g., bleeding, Reye’s syndrome) and transient local symptoms associated with the inhalation of LASAG (e.g., cough, taste alterations, irritation); influenza-related and all-cause mortality documented at follow-up visit #3; adverse events and treatment discontinuation; physical examination and vital signs; symptoms of airway reactivity; pharyngeal symptoms; and clinical laboratory assessments (hematology, biochemistry and blood clotting parameters). The analysis of adverse events, treatment emergent adverse events, and mortality was part of the secondary analysis as defined by the statistical analysis plan (SAP). The trial was registered with EudraCT with identifier number 2012-004072-19.

### Statistical analysis

Statistical significance for the in vitro experiments was evaluated using one-way analysis of variance (ANOVA) followed by Bonferroni’s post hoc comparison tests. A *P*-value < 0.05 indicated a statistically significant difference (*); *P* < 0.01 (**) and *P* < 0.005 (***). The primary analysis was conducted with the MITT population. Only patients in the placebo and the LASAG groups were included in the primary analysis. Each patient was analyzed as assigned by randomization and a *t*-test was conducted. If the assumptions for a *t*-test were significantly violated, a Wilcoxon rank sum test was used instead. To address censored observations, Kaplan-Meier estimates and log rank tests were used. The concrete choice of one of these three test methods was made before un-blinding the data and conducting the data analysis. Sensitivity analyses to support the primary result used the PP population, consisting of all patients without major protocol deviations as defined in the SAP. If one of the tests (Wilcoxon, t-test or log rank) was used, the other test statistics were computed for further sensitivity analyses assessing the impact of this choice. As outlined in the SAP, the primary analysis used one-sided tests with an alpha level of 2.5%. In an additional interpretation of results, a significance level of 5% (one-sided) was used to support the findings. This significance level is justified because the study was declared as a phase II study. In such studies, the results do not represent proof but rather the possibility of efficacy.

## Results

### LASAG acts via inhibition of IKK-mediated NF-**κ**B activity and does not cause inhibition of virus-induced MAPK activation

**Fig. 1:**
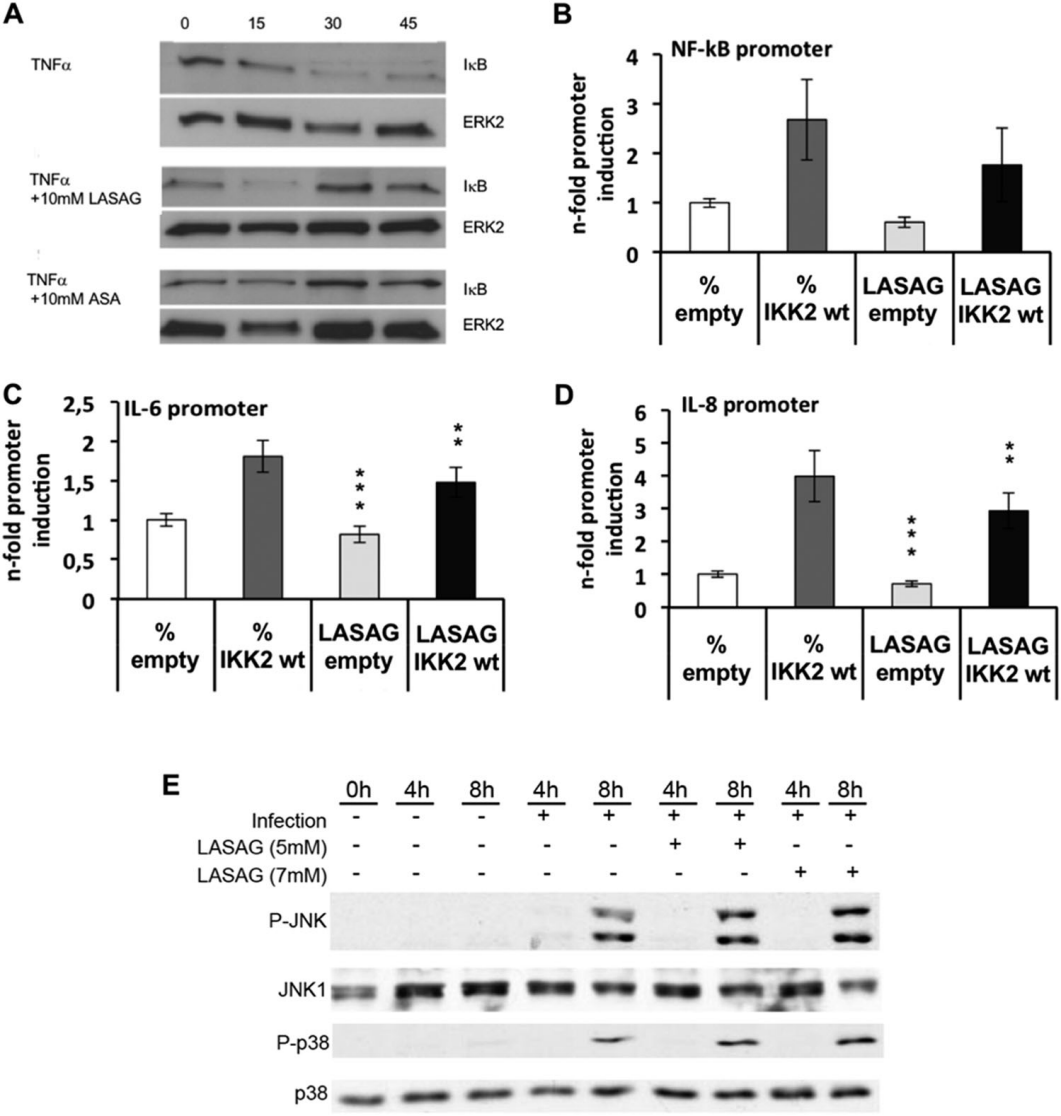
LASAG specifically acts via inhibition of IKK-mediated NF-κB activation and has no impact on virus-induced MAPK activation. **a** Activation of the NF-κB signaling pathway in A549 cells via TNF-α leads to degradation of IκB. IκB degradation and a NF-κB activation are inhibited after treatment of A549 cells with either 10 mM LASAG (BAY 81–8781) or 10 mM ASA. ERK2 represents the loading control. **b**, **c**, **d** LASAG inhibits IKK-mediated transcriptional activation of NF-κB-dependent promoters. A549 cells were transfected with plasmids carrying a NF-κB-specific promoter element in front of a luciferase gene **b** or the promoter constructs of NF-κB-dependent genes IL-6 **C** and IL-8 **d**. Cells were co-transfected with either empty vector or a plasmid expressing a wt form of IKK2 that is active upon overexpression. At 16 h post-transfection, cells were treated with solvent or 5 mM LASAG for an additional 6 h. Cells were then lysed and promoter activity was determined by measuring luciferase activity. The results show the mean of three independent experiments. *P < *0.05 = *; *P < *0.01 = **; *P < *0.005 = ***. **e** LASAG does not have non-specific effects on virus-induced activity of mitogen-activated protein kinases (MAPK) JNK and p38. A549 lung epithelial cells were either left uninfected (lanes 1–3) or were infected with IAV A/FPV/Bratislava/79 (H7N7) (MOI = 5) for 4 or 8 h, respectively (lanes 4–9). Infected cells were either left untreated (lane 1–5) or treated with either 5 mM (lanes 6 and 7) or 7 mM LASAG (lanes 8 and 9) immediately after infection. Cells were then lysed, and protein lysates were separated by PAGE and blotted onto nitrocellulose membranes. Membranes were then incubated with antibodies against phosphorylated active forms of MAPKs JNK and p38. Pan-JNK1 and p38 blots served as loading controls

First, we investigated whether LASAG shows the same pattern of NF-κB inhibition as ASA. As a prerequisite for NF-κB activation, IKK promotes the ubiquitinylation and consequently degradation of IκB (Supplementary Fig. S1). Stimulation of A549 cells with 20 µg/mL TNFα leads to the activation of NF-κB and consequent degradation of ΙκΒ at 30 and 45 min after stimulation (Fig. 1a upper panel). This degradation was inhibited when TNFα-stimulated A549 cells were treated with either 10 mM LASAG (Fig. 1a middle panel) or 10 mM ASA (Fig. 1a lower panel). Thus, both LASAG and ASA can inhibit NF-κB activation. To demonstrate that LASAG specifically inhibits NF-κB induction by IKK in the absence of any extra-cellular or systemic stimulus, we expressed a wild-type (wt) form of IKK2 that is active in cells when overexpressed, together with luciferase reporter gene plasmids carrying either a specific NF-κB promoter element (Fig. 1b) or promoter regions of NF-κB-dependent genes such as IL-6 and IL-8 (Fig. 1c,d). Sixteen hours after transfection, an effective antiviral concentration of 5 mM LASAG was added for six further hours. Although the inhibitor remained on the cells only for these six hours, we observed a partial suppression of the activity of all promoter elements to a similar extent, indicating that LASAG directly affects IKK2-induced NF-κB activity.

To further confirm that LASAG does not have an non-specific effect on the activity of other kinases that are known to influence virus replication, we analyzed the impact of LASAG treatment on virus-induced activation of mitogen-activated protein kinases JNK and p38 (Fig. 1e). These MAPKs are commonly co-activated by stimuli that activate NF-κB, and both p38 and JNK have been found to play a role in various aspects of IAV replication^34–36^. Figure 1e shows that p38 and JNK are readily activated upon infection with IAV 8 h post-infection but that LASAG had no non-specific effect on these kinases.

### LASAG inhibits progeny influenza virus production and protects mice from a lethal challenge

**Fig. 2:**
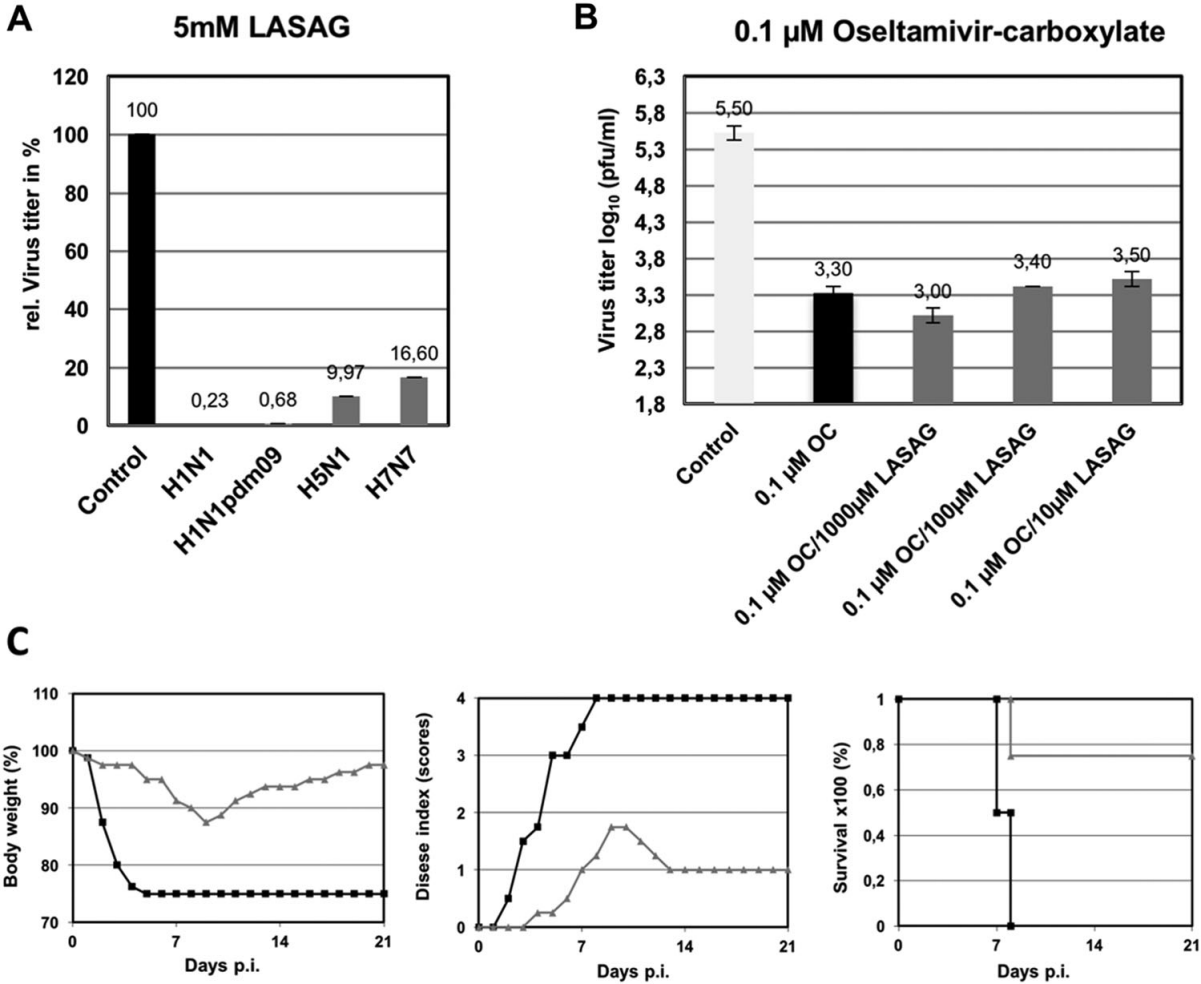
Antiviral activity of LASAG in vitro and in mice. **a** A549 cells were infected with A/Puerto Rico/8/1934 (H1N1) (MOI: 0.01; A/Regensburg/D6/2009 (H1N1pdm09) (MOI: 0.001), A/Mallard/Bavaria/1/2006 (H5N1) (MOI: 0.001), or A/FPV/Bratislava/79 (H7N7) (MOI: 0.001). At 30 min after infection, cells were treated with 5 mM LASAG, and 24 h later, virus titers were determined from the supernatants. The results are presented as percent virus titers relative to infection without LASAG treatment. **b** A549 cells were infected with A/Mallard/Bavaria/1/2006 (H5N1) (MOI: 0.001). At 30 min after infection, cells were treated with either 0.1 µM oseltamivir carboxylate (OC) alone or in combination with either 1000 µM, 100 µM, or 10 µM LASAG. Twenty-four hours later, virus titers were determined from the supernatants. The results are presented as virus titers in log_10_ pfu/ml. **c** Eight-week-old C57BL/6 mice (four per group) were anesthetized with ketamine/rompun and infected with 1.5 × 10^5^ PFU (5 × MLD_50_) of the influenza virus strain A/FPV/Bratislava/79 (H7N7). Starting 1 h prior to infection, mice received twice-daily treatment with 10% LASAG (gray lines) or solvent (black lines) via inhalation for five days. Bodyweight and clinical symptoms were monitored daily over an observation period of 21 days

Next, we investigated the antiviral potential of LASAG to inhibit the replication of various influenza virus strains. As shown in Fig. 2a, LASAG can inhibit progeny production of representative avian and human influenza virus strains. Inhibition levels of more than 99% were observed against PR8 (Fig. 2a; H1N1) and the pandemic H1N1 strain (Fig. 2a; H1N1pdm09). The level of progeny virus production inhibition was 90% for avian strain H5N1 (Fig. 2a; H5N1) and 83% for FPV (Fig. 2a; H7N7).

The general aim of the present study was to investigate the antiviral potential of LASAG in a phase II clinical study in hospitalized patients. A study with only placebo as the treatment in the control group is not ethically feasible. It was a prerequisite of this study that all patients receive SoC treatment, and most of them received the neuraminidase inhibitor Tamiflu®. Thus, we combined the two compounds and used them in sub-optimal concentrations to investigate whether a combined treatment would lead to poorer results than standard of care alone, or to a synergistic effect in cell culture. When influenza virus-infected A549 cells were treated with a sub-optimal oseltamivir concentration of 0.1 µM, an approximately 2 log_10_ reduction in virus titers was observed (Fig. 2b). This reduction was maintained when oseltamivir was combined with three different concentrations (1000 µM, 100 µM, and 10 µM) of LASAG. These results indicate that LASAG does not interfere with the antiviral potential of oseltamivir.

Next, we investigated the antiviral potential of inhaled LASAG in mice infected with a 5 × MLD_50_ of the severe H7N7 strain FPV. Treatment with LASAG (10%) was administered twice daily with a special inhalation device. LASAG protected 3 out of 4 mice, whereas all control animals died by day 8 post-infection (Fig. 2c; left panel) due to a drastic reduction in body weight (Fig. 2c; right panel) and severe influenza-specific symptoms (Fig. 2c; middle panel). These kinds of experiments in mice were further performed in a large number of animals and with various strains and LASAG concentrations. Moreover, pharmacokinetic studies were performed^25^. The results obtained provided the basis for a phase I toxicology study in humans (unpublished data).

### Recruitment and description of eligible patients for the phase II study

**Fig. 3:**
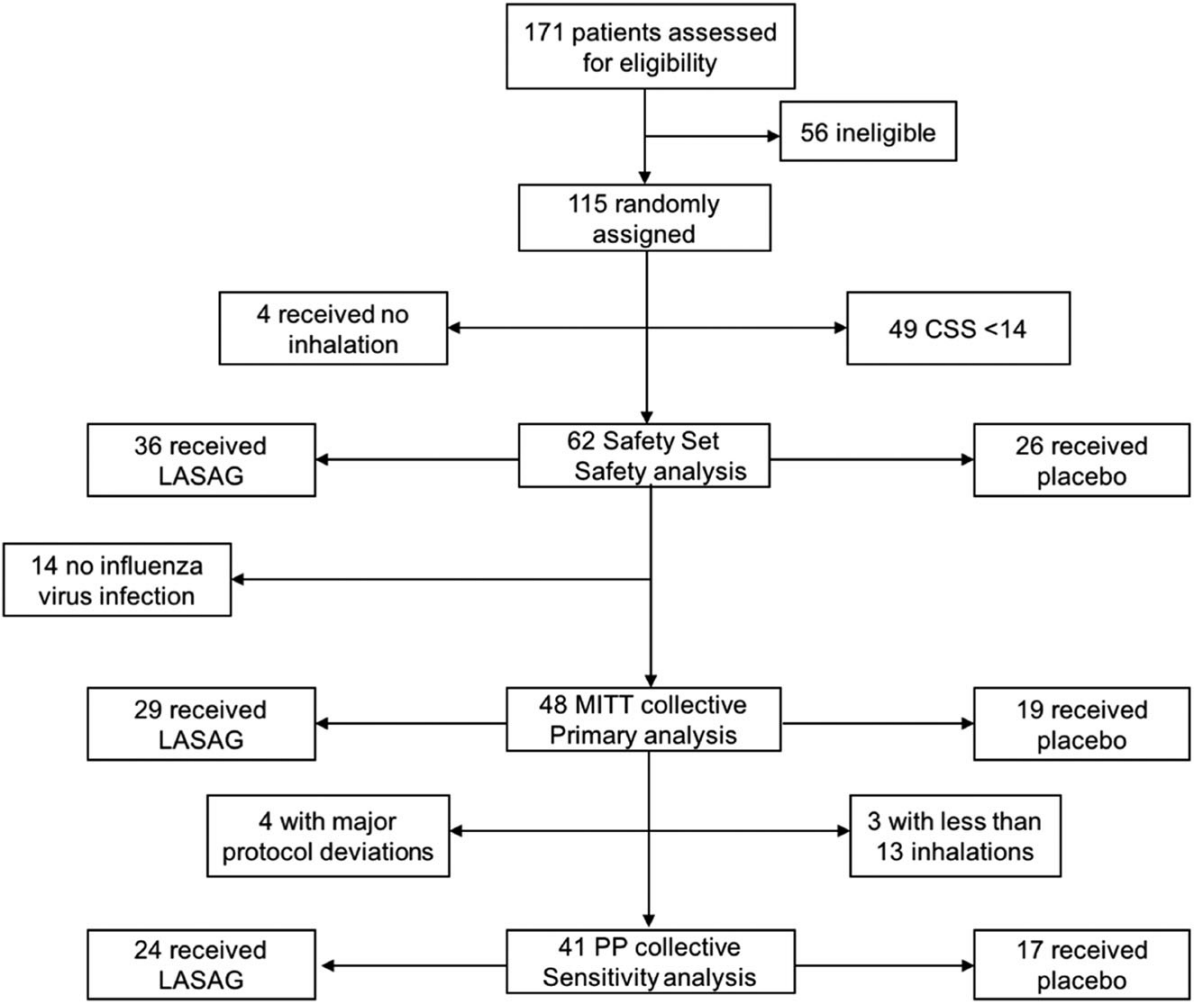
Study population flow chart. Breakdown of patient allocation to different populations

A total of 171 hospitalized patients were screened, of whom 115 were randomized. Fifty-six patients were ineligible because they either did not fulfill all inclusion criteria or they met one or more of the exclusion criteria (e.g., reported onset of illness >120 h; the presence of uncontrolled comorbidities, as outlined in the exclusion criteria). Forty-nine patients had a CSS < 14 and 4 received no inhalation. Of the remaining 62 patients, 36 received LASAG and 26 received placebo. No influenza virus was detectable in 14 patients. The remaining 48 patients constituted the MITT population, of whom 29 received LASAG, and 19 received placebo. Patients in the MITT population who received at least 13 of the 15 inhalations and had no major protocol violations were included in the PP population. Of these, 24 patients received LASAG and 17 patients received placebo (Fig. 3).

The gender distribution was balanced across the two treatment groups (Table 1). A slight gender imbalance was noted in the overall MITT population (59.5% female, 41.5% male). The age distribution was also balanced (average age 41.8 years in the LASAG group and 45.0 years in the placebo group). The distribution of ethnic origin was similar between the two treatment groups. A total of 76% of the patients in the LASAG group and 84% in the placebo group identified their race as European. Of the patients, 24% (LASAG) and 16% (placebo) identified as Hispanic or Latino. The distribution of CSS was also similar between the two groups (LASAG: 16.3, placebo: 16.6). Influenza symptoms (Table 2) and descriptive statistics for various numeric parameters (Table 1) at baseline/screening were collected from the MITT population and showed no obvious differences between the LASAG and placebo groups.

**Table 1:** Baseline characteristics of the modified intention to treat population

	LASAG (*n* = 29)	Placebo (*n* = 19)
Sex		
Male	12 (41%)	8 (42%)
Female	17 (59%)	11 (58%)
Age (years)	41.8 (36.5–47.0)	45.0 (38.9–51.0)
Ethnic origin		
Not Hispanic or Latino	22 (76%)	16 (84%)
Hispanic or Latino	7 (24%)	3 (16%)
Composite symptom score	16.3 (15.7–17.0)	16.6 (15.5–17.7)
Weight (kg)	74.6 ± 15.8	70.8 ± 15.7
Body temperature (°C)	38.5 ± 0.3	38.6 ± 0.3
Heart rate (bpm)	91.1 ± 8.4	91.8 ± 8.8
Systolic blood pressure (mmHg)	117.7 ± 13.3	123.2 ± 21.0
Diastolic blood pressure (mmHg)	72.1 ± 10.8	71.3 ± 14.5
Oxygen saturation (%)	95.3 ± 2.8	95.1 ± 2.6

**Table 2:** Influenza symptoms of the MITT population at baseline/screening

Item	Treatment	Not present		Mild		Moderate		Severe		Not answered	
		*N*	%	*N*	%	*N*	%	*N*	%	*N*	%
Stuffed nose	LASAG	0	0.0	5	10.4	17	35.4	7	14.6	0	0.0
	Placebo	0	0.0	2	4.2	10	20.8	7	14.6	0	0.0
Sore throat	LASAG	0	0.0	0	0.0	20	41.7	9	18.8	0	0.0
	Placebo	0	0.0	3	6.3	9	18.8	7	14.6	0	0.0
Cough	LASAG	0	0.0	1	2.1	6	12.5	21	43.8	1	2.1
	Placebo	0	0.0	1	2.1	7	14.6	11	22.9	0	0.0
Aches, myalgia	LASAG	0	0.0	4	8.3	13	27.1	11	22.9	1	2.1
	Placebo	1	2.1	3	6.3	5	10.4	10	20.8	0	0.0
Fatigue	LASAG	0	0.0	0	0.0	13	27.1	15	31.3	1	2.1
	Placebo	0	0.0	1	2.1	4	8.3	14	29.2	0	0.0
Headaches	LASAG	1	2.1	0	0.0	20	41.7	8	16.7	0	0.0
	Placebo	1	2.1	1	2.1	11	22.9	6	12.5	0	0.0
Fever	LASAG	0	0.0	2	4.2	21	43.6	6	12.5	0	0.0
	Placebo	0	0.0	0	0.0	9	18.6	10	20.3	0	0.0

### Primary outcome of the study: time to alleviation of clinical symptoms

**Fig. 4:**
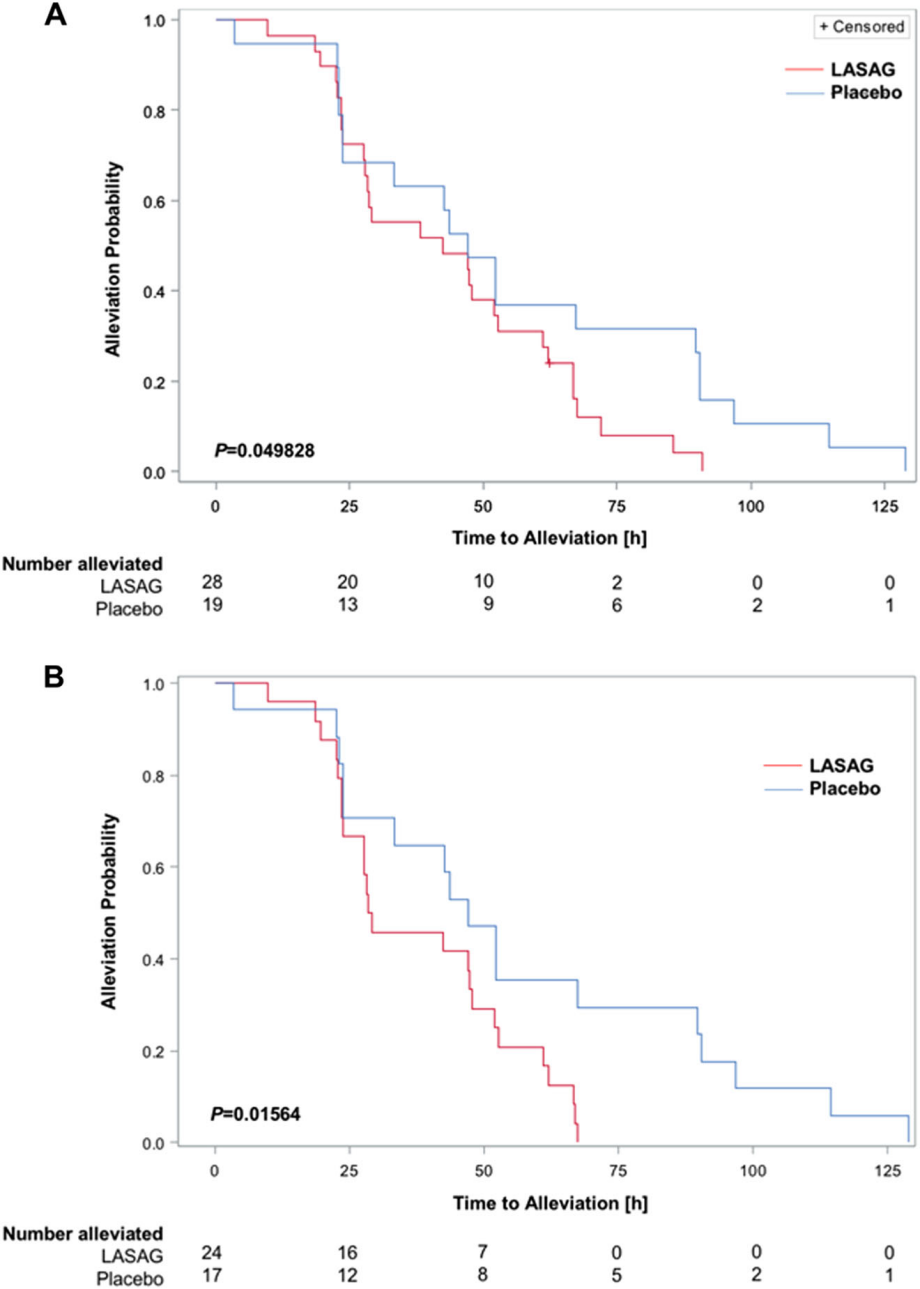
Kaplan-Meier estimation of time to clinical symptom alleviation. **a** The MITT subset consisted of patients with RT-PCR-confirmed influenza and CSS ≥ 14. As censoring occurred within the population, Kaplan–Meier estimates and the log rank test were used for primary hypothesis testing. The *P*-value obtained with the log-rank test was *P* = 0.049828 (in favor of LASAG) **b** Per protocol analysis of patients with RT-PCR confirmed influenza and CSS ≥ 14. The difference was statistically significant based on a log rank test with *P* = 0.01564 (in favor of LASAG)

Time to alleviation of clinical symptoms was defined as the primary outcome. The primary analysis used the MITT population of patients with severe influenza (CSS ≥ 14), RT-PCR confirmed influenza, and at least one inhalation of study drug or placebo. One LASAG patient did not achieve alleviation of symptoms at the end of the study, including the follow-up period. This implied that there was no significant difference between the treatment groups (*P = *1.00 exact test). The mean time to alleviation of the remaining patients in the LASAG group (43.0 h) was lower than that in the placebo group (56.2 h). Thus, patients in the placebo group needed approximately 30% more time for symptom alleviation than those in the LASAG group. Details of the time to symptom alleviation for the 47 patients who recovered are presented in Table 3. The differences in time to symptoms alleviation between the two groups were analyzed using a one-sided Satterthwaite t-test and were not statistically significant (*P* = 0.08 in favor of LASAG). As censoring occurred within the study population, Kaplan-Meier estimates and log rank tests were used for primary hypothesis testing. The Kaplan–Meier curves presented in Fig. 4 intersected only within the first 24 h and showed a faster alleviation of symptoms in the LASAG group thereafter. The difference in time to symptom alleviation between the two groups was analyzed using the log rank test and was statistically significant (*P* = 0.049828 in favor of LASAG) (Fig. 4a). Within the PP population, the mean time to symptom alleviation in the LASAG group (38.2 h) was shorter than in the placebo group (56.1 h). The difference in time to symptom alleviation between the two groups in the PP population was statistically significant (*P* = 0.0365 (one sided Satterthwaite *t*-test)). As in the previous analysis, the Kaplan-Meier curves crossed each other until 24 h of observation in the PP subset (Fig. 4b), after which the LASAG group showed a faster time to symptom alleviation. This difference was also statistically significant (log rank test; *P* = 0.01564, one-sided in favor of LASAG).

**Table 3:** Time to alleviation of influenza symptoms and time to alleviation of clinical signs (alleviated patients)

		Patients (*n*)	Mean	95% CI
Time to alleviation of influenza symptoms				
MITT collective^a^				
	LASAG	28	43.03	34.9–51.2
	Placebo	19	56.21	40.1–72.3
PP collective^b^				
	LASAG	24	38.27	31.1–45.5
	Placebo	17	56.15	39.0–73.3
Time to alleviation of clinical signs				
MITT collective^c^				
	LASAG	28	24.92	17.6–32.2
	Placebo	19	44.10	27.4–60.8

^a^one-sided Satterthwaite *t*-test *P* = 0.08 in favor of LASAG

^b^one-sided Satterthwaite *t*-test *P* = 0.0365 in favor of LASAG

^c^one-sided Satterthwaite *t*-test *P* = 0.00246 in favor of LASAG

### Secondary outcome of the study: alleviation of clinical signs

Time to alleviation of clinical signs was one of the secondary outcomes. As mentioned above, one LASAG patient did not achieve alleviation of symptoms by the end of the study, including the follow-up period. Thus, a total of 47 patients achieved alleviation of clinical signs at the end of the study (incl. follow-up): 28 (96.55%) in the LASAG group and 19 (100%) in the placebo group. The difference between proportions was not statistically significant (*P* = 1.00, exact test). The mean time to alleviation was longer for the placebo group (44.10 h) compared to the LASAG group (24.92 h) (Table 3). This difference was statistically significant (one-sided *t*-test *P* = 0.0246 in favor of LASAG). Other secondary outcomes included decrease in CSS during treatment and routine daily activity scores (DAS). Improvement in both parameters was more pronounced in the LASAG group compared to the placebo group; however, the differences were not statistically significant.

### LASAG treatment reduces viral RNA

Since no quantitative values for viral load were obtained, a qualitative analysis of viral load prior to the eight inhalation treatments was performed using RT-PCR to determine the infection status of the patients (negative/positive). The test was successfully performed in 45 out of the 48 patients in the MITT population; results are shown in Table 4. The chi-squared test showed no statistically significant difference between the LASAG and placebo groups (two-sided Chi-squared test: *P*-value = 0.26). The proportion of patients with negative RT-PCR after eight inhalation treatments was slightly larger in the LASAG group (63% LASAG vs. 57% placebo). It is worth noting that in a subgroup with a higher severity of influenza (CSS ≥ 17), the difference in negative RT-PCR for viral RNA was more pronounced (50% LASAG vs. 29% placebo).

**Table 4:** Presence of influenza virus specific RNA

LASAG		Placebo		Total
*N*	%	*N*	%	*N*
18	69.2	10	52.6	28
8	30.3	9	47.4	17
26	100.0	19	100.0	45

### No severe adverse events occurred after LASAG treatment

A total of 19 adverse events (AEs) affecting 10 patients were reported: 14 adverse events in the LASAG group and 5 in the placebo group. The number of affected patients was 6 (50%) in the LASAG group and 3 (33%) in the placebo group. There was no significant difference between the placebo and LASAG groups (chi-squared test: *P* = 0.45). The grading of severity yielded very similar results for both treatments (LASAG and placebo). The incidence of AEs graded as mild was 57% (8 AEs) in the LASAG group and 40% (2 AEs) in the placebo group. Severe adverse events occurred only in the placebo group. Both severe adverse events consisted of odynophagia, with two distinct episodes occurring in a single patient. Only one adverse event was graded as unresolved. The affected patient (LASAG) suffered from anemia (moderate intensity, not related to treatment, not serious). All AEs in the placebo group were classified as resolved (Supplementary Table S1).

## Discussion

As early as 2007, we were able to demonstrate that ASA has direct antiviral activity against influenza virus by inhibiting the NF-κB signaling pathway. Activity of this pathway is required for efficient influenza virus replication^13^. In mice, the amount of ASA required in the lung could not be provided via an oral administration route. Inhalation of ASA was not suitable for further pre-clinical and possibly clinical development due to the acidic character of the drug. In contrast, administration of aerosolized ASA, formulated as LASAG was well tolerated in mice and demonstrated antiviral effects in cell culture (Fig. 2a, b) and in a mouse model (Fig. 2c).

A Phase I clinical trial demonstrated that inhalation of LASAG was well tolerated and did not lead to adverse events (unpublished data). Thus, LASAG was thought to be suitable for testing of antiviral activity in patients with severe influenza. Administration of aerosolized LASAG resulted in a significantly faster alleviation of influenza symptoms compared to placebo in patients with severe influenza (CSS ≥ 14, receiving at least one inhalation) and in the PP subset of these patients, who received at least 13 inhalations. As all patients received SoC treatment, aerosolized LASAG in hospitalized patients suffering from severe influenza is superior to the standard of care. At the present, there is an ongoing debate regarding whether SoC treatment in addition to treatment with the compound under investigation will result in a proper estimation of effects, but placebo-controlled randomized controlled trials (RCTs) without SoC treatment are considered to be ethically unfeasible.

The primary analyses and all significant secondary efficacy results were in favor of LASAG. These results represent a paradigm change in anti-influenza virus therapy. This is the first clinical study showing that targeting a cellular signaling pathway exploited by the virus, rather than targeting the virus directly, can be effective in severe influenza.

The beneficial impact of LASAG treatment on inflammatory reactions is well known^37,38^. The present study demonstrates an effect of LASAG treatment on the viral load in patients, although it did not reach statistical significance. This is consistent with the findings of earlier pre-clinical studies, which demonstrated a direct effect of Aspirin® treatment on virus titers in the lungs of influenza virus-infected mice^13^. At high concentrations, LASAG inhibits the translocation of NF-κB from the cytosol to the nucleus by specifically inhibiting the upstream kinase IKK. Numerous studies have shown that NF-κB activation is a prerequisite for efficient influenza virus replication^11–16,39^.

Because the same concentration range is needed to both inhibit viral replication and block NF-κB, we hypothesize that LASAG’s antiviral action is due to NF-κB inhibition. This concentration level is much higher than is required for LASAG’s well-documented COX inhibition^13^, which could explain why COX inhibitors such as indomethacin are ineffective at blocking influenza virus replication^14^. Oral treatments are unable to reach systemic concentrations of LASAG sufficient to inhibit NF-κB. Only aerosolized delivery of LASAG directly to the lung reaches effective concentrations resulting in antiviral activity, further supporting the hypothesis that NF-κB is the target.

Activated NF-κB initiates the transcription of TRAIL and FasL, which have been shown to enhance influenza virus propagation in autocrine and paracrine pathways^11,40^. LASAG treatment blocks nuclear export of viral genomes and thereby the release of mature influenza virus, which leads to more rapid decline in viral shedding and decreased risk of viral spread. The cellular target of LASAG and data from *in vitro* experiments suggest that the emergence of resistance will be extremely unlikely^25^.

Although all patients received SoC treatment (most often Tamiflu®), LASAG appears to be superior in reducing viral load compared to SoC treatment alone. A limitation of the study is that the absence of viral RNA in LASAG-treated patients did not reach statistical significance; the reduction in viral load was nevertheless impressive. In a follow-up study, we will focus on viral load more intensively.

The primary endpoint efficacy analysis yielded a significant result in favor of LASAG. The safety analysis indicated no significant differences between the treatment groups. Only one serious adverse event occurred, with no death. The analysis of these patients suffering from serious influenza supports the applicability of inhaled LASAG in treating severe influenza. This study thus represents a first PoC for the use of cellular signaling to treat severe influenza virus infection. A multicenter phase III trial is the next step to confirm the clinical results observed in this PoC study.

## Electronic supplementary material

Supplementary Figure S1

Supplementary Table 1

Supplementary Figure 1 legend
