# Supplementary figures and images for: Targeting intracellular signaling as an antiviral strategy: aerosolized LASAG for the treatment of influenza in hospitalized patients
Source: Emerg Microbes Infect. 2018 Mar 7;7:21. doi: 10.1038/s41426-018-0023-3 (PMC5841227; doi:10.1038/s41426-018-0023-3)

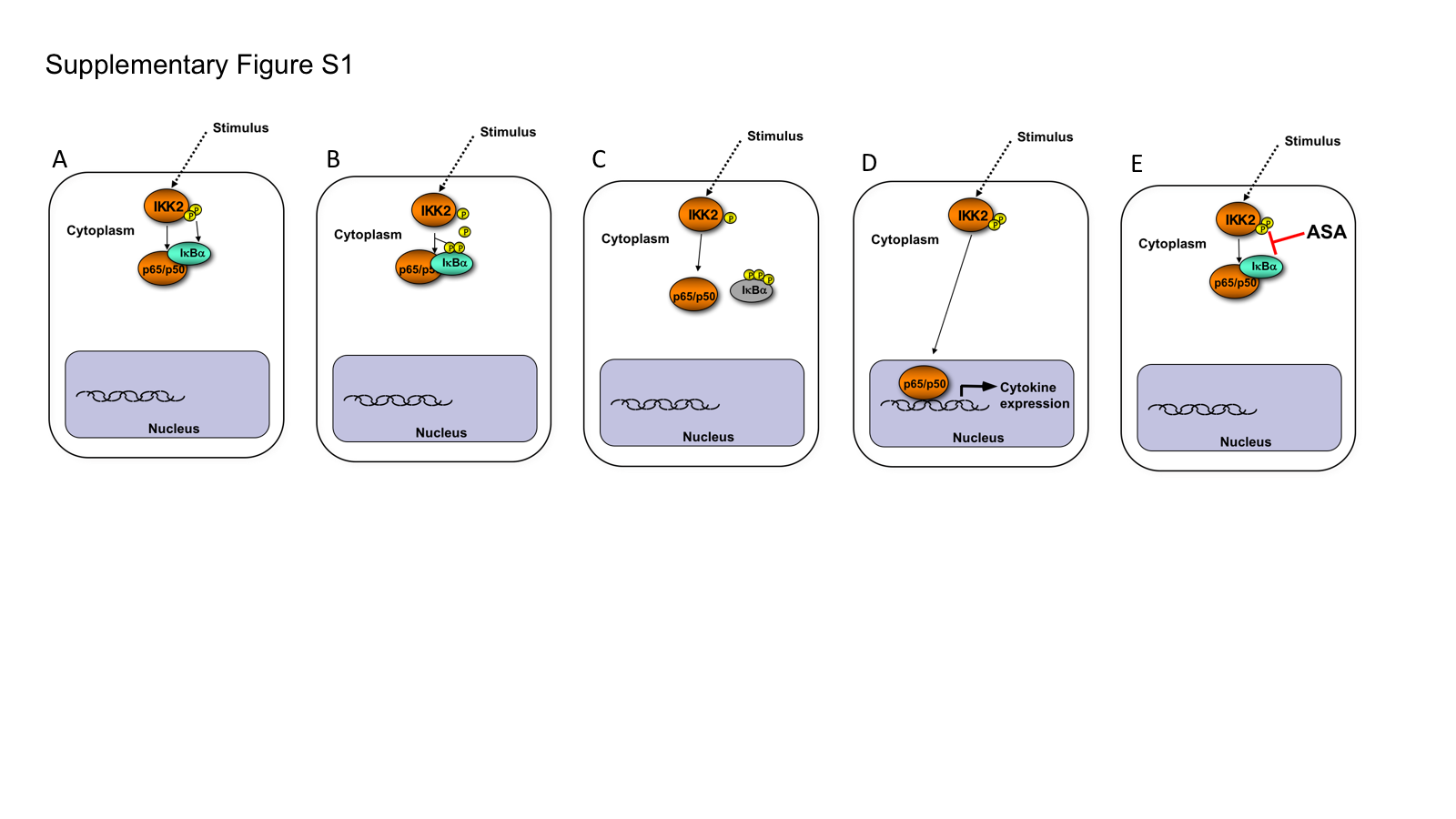

Supplement: Supplementary file 1 — Supplementary Figure S1 [file 41426_2018_23_MOESM1_ESM.tif]
