# Supplementary material for: Targeting intracellular signaling as an antiviral strategy: aerosolized LASAG for the treatment of influenza in hospitalized patients
Source: Emerg Microbes Infect. 2018 Mar 7;7:21. doi: 10.1038/s41426-018-0023-3 (PMC5841227; doi:10.1038/s41426-018-0023-3)
Supplement: Supplementary file 2 — Supplementary Table 1 [file 41426_2018_23_MOESM2_ESM.pdf]

Supplementary Table S1

Number of adverse events (AE) by preferred term (N = count of AEs)

Preferred term (abbreviated)	LASAG N	LASAG Rate %	Placebo N	Placebo Rate %	Total N	Total Rate %
Anaemia	1	7.14	0	0.00	1	5.26
Bronchitis	1	7.14	0	0.00	1	5.26
Constipation	2	14.29	0	0.00	2	10.05
Cough	1	7.14	0	0.00	1	5.26
Headache	0	0.00	0	0.00	0	0.00
Hyperhidrosis	0	0.00	1	20.00	1	5.26
Hypertension	1	7.14	0	0.00	1	5.26
Insomnia	0	0.00	0	0.00	0	0.00
Lower respiratory tract	2	14.29	0	0.00	2	10.05
Meningism	0	0.00	1	20.00	1	5.26
Nausea	2	14.29	1	20.00	3	15.78
Odynophagia	0	0.00	2	40.00	2	10.05
Pharyngitis	1	7.14	0	0.00	1	5.26
Throat irritation	1	7.14	0	0.00	1	5.26
Vomiting	2	14.29	0	0.00	2	10.05
Total	14	100.00	5	100.00	19	100.00

Grading of severity of adverse events (N = count of AEs)

Severity grade	LASAG		Placebo		Total	
	N	Rate %	N	Rate %	N	Rate %
Mild	8	57.14	2	40.00	10	52.63
Moderate	6	42.86	1	20.00	7	36.84
Severe	0	0.00	2	40.00	2	10.05
Total	14	100.00	5	100.00	19	100.00

Outcome of adverse events (N = count of AEs)

	LASAG		Placebo		Total	
Outcome	N	Rate %	N	Rate %	N	Rate %
Resolved	13	92.86	5	100.00	20	95.24
Not resolved	1	7.14	0	0.00	1	4.76
Total	14	100.00	5	100.00	19	100.00
