# Supplementary material for: Targeting intracellular signaling as an antiviral strategy: aerosolized LASAG for the treatment of influenza in hospitalized patients
Source: Emerg Microbes Infect. 2018 Mar 7;7:21. doi: 10.1038/s41426-018-0023-3 (PMC5841227; doi:10.1038/s41426-018-0023-3)
Supplement: Supplementary file 3 — Supplementary Figure 1 legend [file 41426_2018_23_MOESM3_ESM.docx]

**Figure legend S1** Simplified illustration of the mechanisms of how ASA inhibit NF-κB signal pathway via IKK2. NF-κB is a heterodimer of the p65 and p50 protein. As a prerequisite for NF-κB activation IKK promotes the ubiquitinylation and consequently degradation of IκBα (inhibitor of kappaB alpha). **(A)** An external stimulus (e.g. TNFα stimulation, influenza virus infection) leads to the activation of IKK2 (inhibitor of kappaB kinase 2). **(B)** This leads to phosphorylation of IκBα, which is bound to NF-κB. **(C)** Phosphorylation of IκBα results in ubiquitinylation and consequently degradation and therefore in release of NF-κB. **(D)** Consequently NF-κB can migrate to the nucleus and as a transcription factor it leads to expression of proteins, which are needed for influenza virus infection (see introduction). **(E)** By inhibition of IκBα-phosphorylation via ASA, NF-κB remains inactive in a complex with IκBα and can’t migrate into the nucleus.
